# The effect of Charlson Comorbidity Index, race, and surgical complications on postoperative knee outcomes after total knee arthroplasty

**DOI:** 10.1007/s00402-025-05867-z

**Published:** 2025-04-19

**Authors:** Marcel G. Brown, Ayobami S. Ogunsola, Matthew S. Gwilt, Davis Brady, Leslie Granados, John S. Shields, Xue Ma

**Affiliations:** 1 Atrium Health Wake Forest Baptist, Department of Orthopaedic Surgery and Rehabilitation, 1 Medical Center Blvd, Winston-Salem, NC 27157 USA; 2 Wake Forest University School of Medicine, Winston-Salem, USA

**Keywords:** Osteoarthritis, Knee, Arthroplasty, Patient-reported outcome measures (PROMs), Charlson comorbidity index (CCI)

## Abstract

**Introduction:**

Total Knee Arthroplasty (TKA) is the primary definitive treatment for knee osteoarthritis (OA) and has been essential in helping patients reduce knee pain and regain mobility. There is a need to assess whether various factors such as surgical complications from primary TKA, pre and postoperative range of motion (ROM), Charlson Comorbidity Index (CCI), comorbidities other than CCI, and demographics representative of an outpatient TKA population affect patient-reported outcome measures (PROMs).

**Materials and methods:**

Retrospective chart review was performed on 444 patients who underwent TKA at an outpatient surgical facility. Demographics, qualitative, and quantitative measurements were collected at baseline, 4–6 months, and 1-year postoperatively. Patients were stratified by CCI into low (< 2), moderate (2–4), and high (> 4) risk categories. A generalized linear model was used to assess the relationship between time, complications, risk categories, and Knee injury and Osteoarthritis Outcome Score Joint Replacement (KOOS, JR).

**Results:**

Majority of patients were women (58.9%), non-Hispanic white (81.9%), categorized as moderate risk CCI (78.8%), with 22.5% experiencing complications post-TKA. KOOS, JR scores improved over time, with an increase of 18.1 points at 4–6 months and 26.1 points at 1-year post-TKA (*p* < 0.0001). Surgical complications were linked to a decrease of 3.5 points in KOOS, JR scores, whereas patients with high pre-TKA KOOS, JR scores had an increase of 6.4 points after surgery. Patients who identified as African American experienced an average of 4.7 points lower on KOOS, JR than non-Hispanic whites (*p* = 0.0211). High-risk patients (CCI > 4) on average, had higher KOOS, JR scores 12 months after TKA. African Americans and those with surgical complications reported Lower KOOS, JR scores.

**Conclusions:**

TKA improved KOOS, JR scores through one year with the greatest improvement in PROM being in higher-risk patients, those without surgical complications. Patients with surgical complications and/or African American race had a lower average KOOS, JR score.

## Introduction

Osteoarthritis (OA) of the knee is the most common form of OA worldwide, with an estimated prevalence of 22.9% among adults 40 and older, and one of the primary forms of disability in the United States [1]. Generally, management of knee arthritis is primarily nonsurgical, with progression to total knee arthroplasty (TKA) once conservative treatments have been exhausted [2]. TKA is not only the most effective treatment for decreasing pain due to knee OA, but simultaneously increases patient functionality and improves quality of life [3, 4].

One common way to assess patient outcomes after TKA is the Knee injury and Osteoarthritis Outcome Score (KOOS). KOOS is a validated patient-reported outcome measure (PROM) that asks patients 42 questions regarding pain, other symptoms, functions in daily living, function in sport and recreation, and knee-related quality of life. It is a valuable and validated PROM that is scored out of 100, with higher scores indicating less pain or adverse symptoms, improved functionality, and a higher quality of life [5, 6]. The Knee injury and Osteoarthritis Outcome Score Joint Replacement (KOOS, JR) score which measures similar values but does so with only 7 questions. Though abbreviated, the KOOS, JR has been shown to be an equally valid measure of patient outcomes in the United States and abroad [7, 8].

While KOOS, JR is a standardized and valuable measure of assessing TKA outcomes in patients, it is designed not to capture the factors that may influence its score. Most importantly, KOOS, JR Is agnostic to comorbidities that may influence surgical outcomes. Indeed, comorbidities such as hypertension, cancer, and diabetes are known factors that can lead to complications following TKA [9–13]. Further, it is well accepted that the risk of revision surgery is greater in patients with more comorbidities [14–17]. Acknowledging these risk factors, it has become commonplace for surgeons to have strict criteria when choosing who should receive a TKA and to be cognizant of the important factors that influence TKA outcomes. Possible contraindications for a patient to undergo TKA include poor mental health, cardiac disease, respiratory disease, diabetes, smoking, and peripheral vascular disease, given their historically negative impact on surgical outcomes and odds of complications, and ultimately more pressing medical needs [18]. Currently, there are few absolute and relative contraindications to undergoing TKA, and generally, risk factors for surgical complications are challenging to address [19, 20]. In general, as a surrogate for measuring comorbidity, surgeons both in orthopaedics and outside of the specialty have relied on the Charleson Comorbidity Index score (CCI) to measure disease burden [21–27]. CCI has provided invaluable information in narrowing comorbidities to a simple score summarizing the burden of disease for a patient and the assessment of the possible surgical outcomes more predictive. In this study, we provide a retrospective analysis of patient-reported KOOS, JR scores following TKA to determine whether a time-dependent relationship exists between these scores and factors such as CCI, surgical complications, and patient demographics.

## Methods

### Data collection

**Fig. 1:**
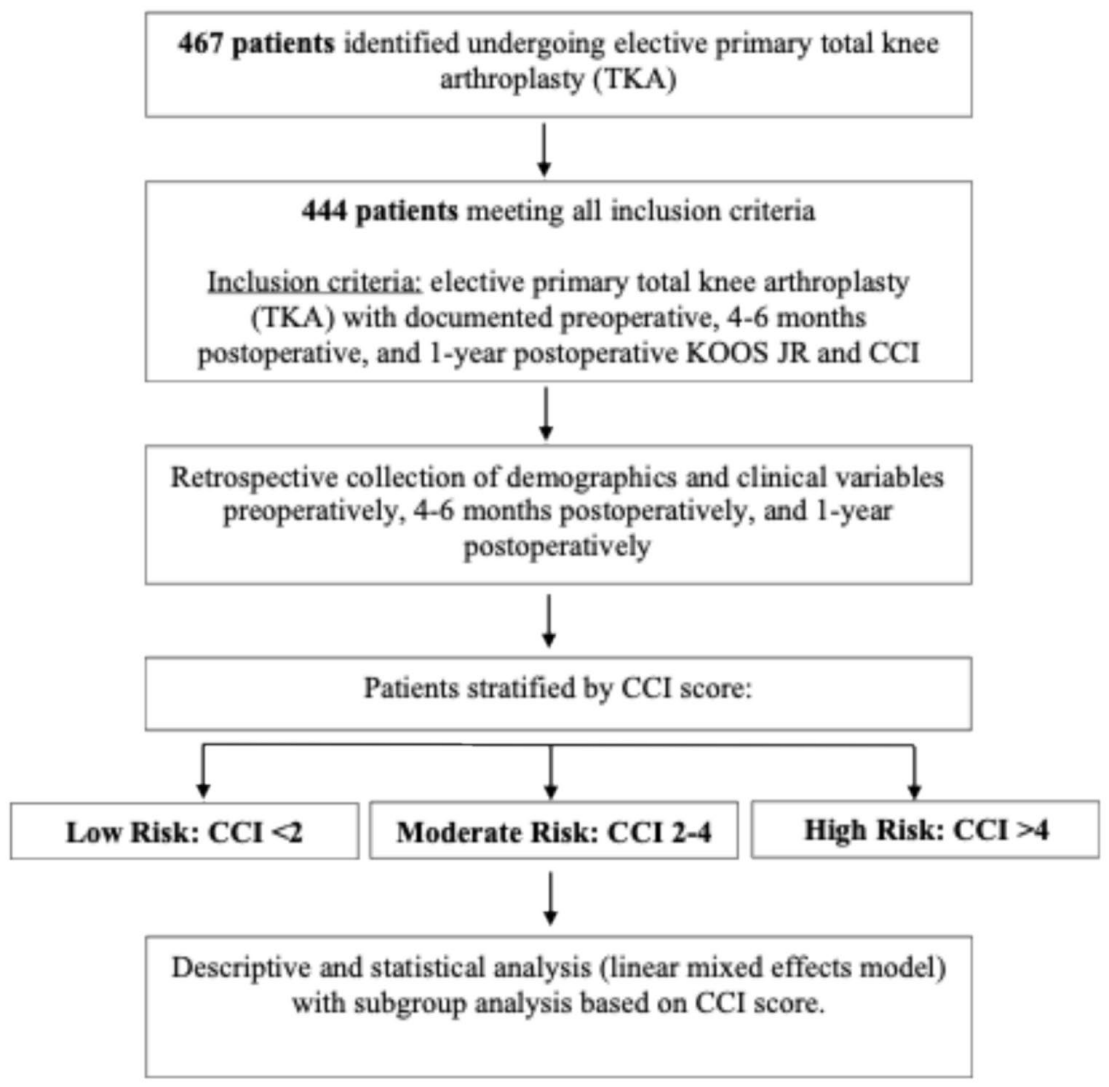
Flow diagram depicting methodology for the study

An institutional review board (IRB) approved retrospective review was obtained from an adult population who underwent elective TKA at an outpatient surgical facility between 2022 and 2024. The study population was followed up for one year after surgery. Pre-surgical demographics, examinations, and laboratory values were collected through standardized chart review. Patients with the following criteria were included in the study - elective primary TKA, documented preoperative KOOS, JR score, 4-6 month postoperative and 1-year postoperative KOOS JR score and CCI score (Fig. 1).

### Variable definitions

Reported demographics including age, sex, and racial identities were recorded. Race was classified as non-Hispanic Whites, Asians, African American, Hispanic, or Others. KOOS, JR scores were used to assess the knee functional outcome of study participants. More specifically, KOOS, JR is scored by adding the raw responses of study subjects (range 0–28) and then converting these raw scores to an interval score ranging from 0 to 100. A score of 0 on the interval scale indicates total knee disability, while a score of 100 represents perfect knee health. KOOS, JR scores were obtained from study participants preoperatively, 4–6 months postoperatively (coinciding with the patient’s first postoperative KOOS, JR score report), and one year postoperatively (coinciding with the patient’s second postoperative KOOS, JR score report). CCI is a standardized score used to predict mortality for patients with various medical conditions. Study subjects received an aggregate CCI score based on the following comorbidities: age, myocardial infarction (MI), congestive heart failure (CHF), peripheral vascular disease (PVD), cerebrovascular accident/transient ischemic attack (CVA/TIA), dementia, chronic obstructive pulmonary disease (COPD), connective tissue disease, peptic ulcer, liver disease, diabetes mellitus, hemiplegia, leukemia, lymphoma, and AIDS (Table 1). CCI scores were used to classify study subjects into low-risk (score < 2), moderate-risk (scores between 2 and 4), and high-risk (scores greater than 4) categories. Data on patients who developed surgical-related complications such as superficial wound infections, deep wound infections, wound dehiscence, excess postoperative pain, deep vein thrombosis (DVT), knee dislocation, and revision TKA were recorded as “Complications” (see Table 2). Surgical side preoperative and postoperative range of motion (flexion and extension) were also documented for study participants.

**Table 1:** CCI scoring system

Clinical Presentation	Assigned Score
Age	
50–59 60–69 70–79 80+	1 2 3 4
MI	1
CHF	1
PVD	1
CVA/TIA	1
Dementia	1
COPD	1
Connective Tissue Disease	1
Peptic Ulcer	1
Liver Disease	1 (mild) 3 (moderate/severe)
Diabetes Mellitus	1 (controlled) 2 (uncontrolled)
Hemiplegia	2
Leukemia	2
Lymphoma	2
AIDS	6

**Table 2:** Postoperative complications

Complications	Number	Percentages (%)
**Surgical related**		
Joint Stiffness Requiring Manipulation Under Anesthesia	19	4.3
Deep Wound Infection	8	1.8
Superficial Wound Infection	18	4.0
Deep Vein Thrombosis	4	0.9
Pulmonary Embolism	2	0.5
Aseptic Loosening	4	0.9
Revision TKA for other Reasons	5	1.1
Postoperative Anemia	1	0.2
Intraoperative Atrial Fibrillation	1	0.2
**Non-Surgical Related**		
Postoperative Myocardial Infarction	1	0.2
Intractable GI symptoms (GI bleed/Vomiting)	2	0.5
Hyponatremia	2	0.5
Postoperative Seizures	1	0.2
Persistent Joint Effusion /Bruising	5	1.1
Hypotension	1	0.2
Drug Reaction (Incision itch/anaphylaxis)	3	0.7
**Total number of complications**	77	17.3
**None**	367	82.7
**Study Sample Size**	444	100

### Statistical analysis

Descriptive statistics were conducted to analyze the distribution of study variables. Percentages were estimated for factor-level variables, and measures of central tendency (mean and standard deviation) were used to describe the distribution of continuous variables. To identify independent predictors explaining the changes in KOOS, JR scores, a generalized linear model (linear mixed effects model) was fitted to the data, modeling KOOS, JR scores as a function of time and other independent variables, while accounting for the repeated measures structure and random variability within subjects. Model residuals were assessed for normality via residual plots and statistics. All statistical analyses were performed using SAS version 9.4 (SAS Institute; Cary, North Carolina).

## Results

**Fig. 2:**
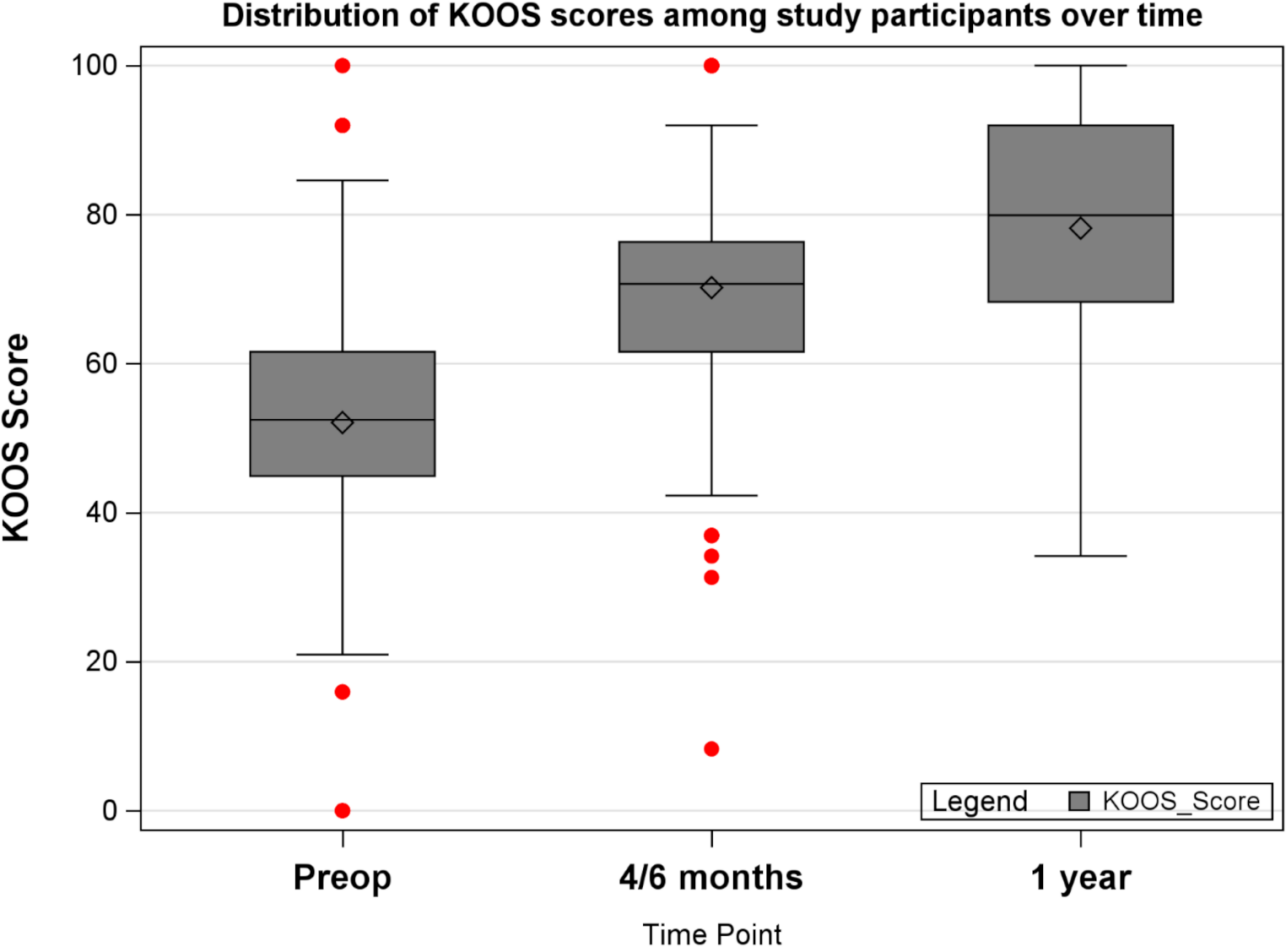
Trend in KOOS, JR scores for patients undergoing total knee arthroplasty, with mean KOOS, JR score improvement shown at 4–6 months, and at one year postoperatively

The total number of study participants in this analysis was 444. The majority of subjects were women (*n* = 260; 58.6%) and non-Hispanic White (*n* = 364; 81.9%). Regarding CCI scores, 14.9% of study participants were considered to have low-risk CCI scores (CCI scores: <2), 78.8% were deemed to be in the moderate-risk category (CCI scores: 2–4), and 6.3% were classified as high-risk prior to TKA (CCI scores: >4). Upon analysis of complications, 17.3% of study participants developed one or more complications post-TKA (Table 3). The distribution of KOOS, JR score at the preoperative, 4–6 month postoperative, and one-year postoperative period is shown in Fig. 2.

**Table 3:** Descriptive statistics of study population

Variables	*N* (%) or Mean (SD)^*^
**Age**	67.2 ± 9.3
**Sex**	
Female Male	260 (58.6) 184 (41.4)
**BMI**	31.2 ± 5.3
**Race**	
Non-Hispanic Whites Asian African American Hispanic Others	364 (81.9) 9 (2.0) 51 (11.5) 7 (1.6) 13 (2.9)
**Preoperative Extension**	2.3 ± 2.9
**Preoperative Flexion**	114.6 ± 9.3
**Postoperative Extension**	2.9 ± 3.8
**Postoperative Flexion**	95.7 ± 13.8
**Length of Hospital Stay**	1.0 ± 0.2
**Serum Creatine**	1.0 ± 0.5
**Preoperative KOOS**,** JR score**	52.1 ± 14.0
**First postoperative KOOS**,** JR score**	70.2 ± 14.2
**Second postoperative KOOS**,** JR score**	78.2 ± 15.9
**Complications**	
No Yes	367 (82.7) 77 (17.3)
**Charlson Comorbidity Index Score**	
< 2 2–4 > 4	66 (14.9) 350 (78.8) 28 (6.3)
**Hyperthyroidism**	
No Yes	423 (95.3) 21 (4.7)
**Hypertension**	
No Yes	149 (33.6) 294 (66.4)

In a longitudinal analysis of knee function following total knee arthroplasty, a mixed-effects model demonstrated significant time-dependent improvements in KOOS, JR scores (Table 4). Patients experienced an average increase of 18.1 points (95% CI: 16.4, 19.8) at 4–6 months postoperatively and 26.1 points (95% CI: 24.4, 27.8) at one year postoperatively, relative to preoperative scores (*p* < 0.0001). The presence of postoperative complications was associated with a notable decrease in KOOS, JR scores, with an average decline of 3.1 points (95% CI: -5.8, -1.1, *p* = 0.013). Conversely, patients with a higher burden of comorbid conditions, as indicated by a CCI score greater than 4, showed a significant increase in KOOS, JR score by 6.3 points (95% CI: 1.7, 11.0, *p* = 0.025) (Table 4). In addition, patients with depression had an average decline of 2.7 points (95% CI: -5.1, -0.3). The model’s intraclass correlation coefficient (ICC) was 21%, indicating a substantial variation in response among individuals (Table 4).

**Table 4:** Linear mixed effects model of KOOS, JR score (outcome) as a function of time and other predictors

Variable	Estimates	Confidence Interval	*P*-value
**Time**			< 0.0001
Preoperative KOOS	Ref		
First Postoperative KOOS	18.1	16.4, 19.8	
Second Postoperative KOOS	26.1	24.4, 27.8	
**Gender**			0.936
Women	Ref		
Men	-0.1	-2.1, 2.0	
**Race**			0.032
Non-Hispanic Whites	Ref		
Asian	4.0	-3.5, 11.5	
African American	-4.5	-7.5, -1.6	
Hispanic	-0.1	-7.8, 7.5	
Others	-1.9	-7.5, 3.7	
**Complications**			0.013
No	Ref		
Yes	-3.1	-5.8, -1.1	
**Hospital Length of Stay**	-1.7	-5.3, 1.9	0.359
**Serum Creatine**	-1.8	-3.9, 0.4	0.106
**Postoperative Extension**	-0.3	-0.5, -0.1	0.026
**Hyperthyroidism**			0.081
No	Ref		
Yes	-4.4	-9.4, 0.6	
**Depression**			0.028
No	Ref		
Yes	-2.7	-5.1, -0.3	
**Hypertension**			0.305
No	Ref		
Yes	1.1	-1.0, 3.2	
**Charlson Comorbidity Index Score**			0.025
< 2	Ref		
2–4	1.3	-1.5, 4.1	
> 4	6.3	1.7, 11.0	

## Discussion

TKA remains as the only definitive treatment for end-stage knee OA to relieve pain, restore knee function and improve function; however, the clinical guidelines for patient eligibility for the procedure remain controversial due to conflicting PROMs [28]. It is critical for orthopedic surgeons to predict outcomes following TKA using pre-surgical risk evaluations in order to anticipate potential complications, evaluate the safety of the surgery, assess a patient’s fitness for surgery, and provide patients with the best, individualized model of care. In our reported patient population at one outpatient surgery center, PROMs continued to improve at 4–6 months and up to 1 year postoperatively. This finding is similar to other studies that report PROMs increasing over the first 6 months post TKA. Interestingly, however, these studies have shown a plateau of PROMs at the 6-month time point with mostly non-significant improvement afterward [29–31]. In contrast, our study showed PROMs continue to improve past the 6-month time point. While seemingly less common, this result is not unique [32, 33]. One study actually reported a decrease in PROMs at 6 months and then an increase at the 1-year mark [33].

Our study shows a correlation between increased KOOS, JR score and a CCI > 4 (Table 3). This correlation indicates that patients with higher pre-operative comorbidities may report experiencing greater benefits from a TKA than those with fewer comorbidities. This is seemingly contradictory as one might expect high-risk patients to experience more adverse health events and, thus, worse surgical outcomes following TKA. On the other hand, this outcome can also be viewed as a more significant improvement in outcomes from baseline for patients with more comorbidities. This result could either reflect good medical optimization or very poor functional status prior to surgery.

It is important to note that we did not conduct an analysis to examine whether higher CCI scores correlate with a greater incidence of complications, as this was outside the scope of our study. Lee et al. established that a higher CCI score (CCI ≥ 4) is a risk factor for readmission for surgical complications and reoperation a year after bilateral TKA [34]. This is a significant result, and similar to our findings, can be related to greater improvements in KOOS scores in patients with more severe baseline OA [29, 35, 36]. Much of the hesitancy related to performing TKA on patients with more significant comorbidities is attributed to the worst outcomes that are often inevitable in this patient population. In one study, the correlation between CCI score and bilateral TKA complications found that patients with high-risk CCI scores (CCI > 4) were more likely to undergo hospital readmission by 90 days, readmission for surgical complications, and readmission by 1 year postoperative [34]. Similarly, a study published by Elmallah et al. reviewed preoperative CCI scores compared to postoperative knee society score (KSS), Short Form 36 (SF-36), and lower extremity activity scale (LEAS) and found that lower CCI scores (thus fewer comorbidities) generally lead to better patient-reported outcomes [37]. Despite these correlations between CCI and complications, there remains a paucity of literature looking at PROMs and, more specifically, KOOS and KOOS, JR scores as stratified by CCI class.

Similar to our findings, PROMs have been shown to increase over time after TKA [29, 30, 33, 38]. Cushnaghan et al. followed TKA patients for six years in a longitudinal study. Comparing them to non-TKA patients, they found that PROMs increased over a 6-year period in the TKA group and decreased in the control groups. Notably, the increase in PROMs was seen regardless of BMI [39]. In specific circumstances, PROMs can decrease over time. Pitta et al. followed patients for 2 and 5 years after TKA and determined that the “other symptoms” category showed a statistically significant reduction in PROMs over time [40]. While this reduction was based solely on one tenant of the KOOS scoring system, it nonetheless indicates that long-term deterioration following TKA is possible.

Similar to our findings, PROMs have been shown to be negatively correlated with TKA complications [41–43]. It is reasonable to suspect that a patient who experiences a complication following a procedure will describe their outcomes more negatively than one who does not. In a study by Larsen et al. the effect of early and late complications during a patellar fracture repair on KOOS, JR score were found to be negatively correlated. Specifically, they found that both early and late complications led to a decreased KOOS, JR score across all five domains [41]. In addition to this, persistent pain following TKA is a frequent occurrence that unsurprisingly can increase post operative dissatisfaction [44]. A meta-analysis performed by Gunaratne et al. found numerous studies demonstrating a correlation between post operative pain and patient dissatisfaction in their TKA surgeries [45].

An additional finding from our study was a significant difference in KOOS, JR scores reported by people who identified as African American. It is well established that minority groups undergo total joint arthroplasty (TJA) at a lower frequency in comparison to white Americans [46–55]. The underutilization of TJA in minority populations has been postulated to be a result of various social determinants of health and socioeconomic factors. Several studies have shown a persistent disparity between minorities and whites, even after controlling for these factors [46, 50, 51]. More recent analysis, has looked at whether postoperative outcomes differ across racial groups following TKAs [47]. In looking at PROMs, a fair amount of data has shown no difference in long-term outcomes between African Americans and whites [32, 56–58]. A systematic review by Goodman et al. in 2016, however, reported that African Americans reported worse pain and worse function 6 months or more after surgery [47]. Notably, Goodman et al. distinguished this finding by publishing another study indicating that pain scores are comparable between blacks and whites who are living in places with little poverty [56]. Two other studies found that KOOS scores were higher for African Americans compared to whites preoperatively, but no clinical or PROM score difference 4 to 8 weeks postoperatively [32] and 1-year postoperatively [58]. Cavanaugh et al. on the other hand found that outcomes 1 and 2 years postoperatively were inferior for black women in comparison to hispanic or white women– these differences were no longer statistically significant after 2 years [57].

In contrast, numerous other studies have indicated significantly different outcomes in African Americans following TKA compared to whites [46, 49, 59–64]. Gungor et al. found that African Americans had an odds of 1.8 in comparison to whites (95% CI: 1.0–3.2) of developing persistent postsurgical pain following TKA [62]. Two other studies found that postoperative complications, such as revision surgery, readmission, discharge to institutional care, and emergency department visits, were significantly more likely to occur in blacks compared to whites [46, 49]. The reason for these disparities is only hypothesized but is likely multifactorial. Many have suggested these differences in postoperative outcomes are due to socioeconomic disparities and differences in access to healthcare between whites and blacks. Hadad et al. used the Area Deprivation Index to assess the influence of socioeconomic status on TKA outcome, finding that low socioeconomic status does indeed correlate with negative postoperative outcomes [65]. This was not a factor that we analyzed in our study. Regardless, it is important for surgeons to look beyond race during TKA selection and consider giving additional support and care to patients who are of a lower socioeconomic status [55]. Social determinants of health, including economic stability and their effect on health went widely unstudied until the late 1960s [66]. Since then, expansive research on social determinants of health has been performed and has shown the tremendous effect these factors can have on the body [67]. There should be no exception in accounting for these factors in the management and optimization of osteoarthritic knee pain and postoperative TKA complications [68–71]. For this reason, we recommend surgeons take an individualized, holistic approach when considering TKA for any patient.

Our study suggests that patients who have a higher CCI may experience greater benefits from TKA than those who have fewer comorbidities. We suspect this change may have been due to better preoperative optimization or a higher level of patient motivation in patients with higher CCI scores. Another possible explanation for this observation is that there may have been unaccounted-for bias, given the fact that the CCI groups were not equal in number or perfectly balanced. Interestingly, the impact of CCI on PROMs is sparse across the literature. One study from 2005 by Harse and Holman suggested that CCI had no impact on health-related quality of life outcomes one year following TKA [72]. On the other hand, Elmallah et al. performed a prospective study with 283 patients looking at how their patients’ CCI scores (0, 1, 2, 3, or 4) affected PROMs at 6 weeks, 3 months, 1 year, and annually until 5 years postoperative. They found that patients with lower CCI scores had significantly higher PROMs at 2 and 5 years postoperative [37]. In further support of these findings, numerous studies have associated higher CCI scores to an increased rate of complications following TKA [73–75]. In this context, the findings in our study remain particularly interesting and should prompt further investigation. It may also be salient to note that one complicating factor in making informed conclusions about the effect of comorbidities on PROMs is the underreporting of comorbidities in the TKA literature. One systematic review by Sinclair et al. found that only 31.4% of TKA studies reported comorbidities in their data analyses [76].

Though we did not directly analyze obesity in the present study, one of the most common comorbidities seen in patients with OA in need of TKA and in particular in the United States, is obesity [14]. There is widespread debate about the efficacy and safety of performing a knee arthroplasty on patients above a BMI of 35 [77–80]. Agarwala et al. assessed whether obesity should be considered a contraindication when performing a knee arthroplasty. For 12 months, they followed 213 patients with BMIs ranging from 30.01 to 39.99 kg/m^2^ who underwent TKA, assigning them a knee society score (KSS) based on their reported outcomes at each follow-up appointment. They found that KSS scores increased from 55.9 to 93.0, and an average functional improvement score ranging from 52.9 to 80.6 at the patients’ final follow-ups [81]. The average increase in KSS scores was also compared to other studies that reported KSS scores between obese and non-obese patients. Most studies indicated there was no significant difference in KSS scores between these two groups [20, 82, 83]. However, a few studies suggest that obese patients report lower KSS scores and often experience patellofemoral symptoms [84–86]. This dichotomy is well established, with some studies finding that BMI is an important consideration [19, 87, 88] and others suggesting that it is not [89–92]. These studies demonstrate the mixed opinions regarding the relationship between obesity and TKA outcomes, highlighting the need for more studies to enhance our clinical decision-making on this front.

Finally, our study showed a negative correlation between KOOS, JR and depression (Table 4). This finding is not surprising and is well established [93, 94]. It is hard to decipher whether depression is exacerbated by the ongoing disability associated with knee osteoarthritis or whether it is due to the pain associated with OA. Regardless, depression undoubtedly effects a patient’s perceived recovery from TKA. Whether or not the surgical procedure of TKA itself is a traumatic event that may worsen depressive symptoms has yet to be proven, but nonetheless it is a contributing factor to a patient’s recovery after undergoing the procedure.

### Limitations

Our study is not without limitations. Most notably, this is a retrospective chart review with follow-up limited to 1 year. Of additional note, our sample population was isolated to one outpatient facility, thus limiting the generalizability of our outcomes. Additional comorbidities other than reported in CCI may play a role in our measured outcomes and are not included in this study. In addition, KOOS, JR score is not without limitations since it is a truncated survey. Nevertheless, it still serves as a validated PROM and one of the most reliable measures of assessing patient outcomes after undergoing TKA [8]. It is also important to note that the low overall complication rate observed in our study is consistent with national benchmarks. Recent data suggest that the incidence of severe adverse events following total joint arthroplasty is approximately 5.7%, whereas the 30-day return to the operating room is 4.8% [95]. Thus, the relatively low complication rate in our study likely reflects the strong performance of our institution in managing all joint cases, further limiting the ability to stratify complications meaningfully.

## Conclusion

Overall, our study demonstrates that comorbid factors that previously may have been thought to negatively affect KOOS, JR scores may actually lend themselves to improved PROMs up to 1 year after TKA. Notably, KOOS, JR scores, on average, increase over time, but decrease with African American race and complications, while they increase in patients with a greater comorbidity burden.

## Data Availability

No datasets were generated or analysed during the current study.
